# Focus on Self-Presentation on Social Media across Sociodemographic Variables, Lifestyles, and Personalities: A Cross-Sectional Study

**DOI:** 10.3390/ijerph191711133

**Published:** 2022-09-05

**Authors:** Gunnhild Johnsen Hjetland, Turi Reiten Finserås, Børge Sivertsen, Ian Colman, Randi Træland Hella, Jens Christoffer Skogen

**Affiliations:** 1 Department of Health Promotion, Norwegian Institute of Public Health, 5015 Bergen, Norway; 2 Centre for Evaluation of Public Health Measures, Norwegian Institute of Public Health, 0473 Oslo, Norway; 3 Department of Research and Innovation, Helse Fonna HF, 5525 Haugesund, Norway; 4 Department of Mental Health, Norwegian University of Science and Technology, 7491 Trondheim, Norway; 5 School of Epidemiology and Public Health, University of Ottawa, Ottawa, ON K1G 5Z3, Canada; 6 Centre for Fertility and Health, Norwegian Institute of Public Health, 0473 Oslo, Norway; 7 Department of Work, Section for Children, Families and Disabled, Social Services and Housing, 5014 Bergen, Norway; 8 Alcohol and Drug Research Western Norway, Stavanger University Hospital, 4036 Stavanger, Norway

**Keywords:** social media, adolescent, self-presentation, social comparison, feedback-seeking

## Abstract

Upward social comparison and aspects of self-presentation on social media such as feedback-seeking and strategic self-presentation may represent risk factors for experiencing negative mental health effects of social media use. The aim of this exploratory study was to assess how adolescents differ in upward social comparison and aspects of self-presentation on social media and whether these differences are linked to sociodemographic variables, lifestyle, or personality. The study was based on cross-sectional data from the “LifeOnSoMe” study performed in Bergen, Norway, including 2023 senior high school pupils (response rate 54%, mean age 17.4, 44% boys). Nine potentially relevant items were assessed using factor analysis, and latent class analysis was used to identify latent classes with distinct patterns of responses across seven retained items. The retained items converged into one factor, called “focus on self-presentation”. We identified three groups of adolescents with a low, intermediate, and high focus on self-presentation. Associations between identified latent classes and covariates were assessed using regression analyses. Being a girl, higher extraversion, lower emotional stability, more frequent alcohol consumption, and having tried tobacco were associated with membership in the high-focus group. These results suggest some characteristics that are associated with a higher focus on self-presentation and that could inform targeted interventions.

## 1. Introduction

Social media are widely used, and the most popular social media platforms have up to 2.9 billion active users [1]. Social media “employ mobile and web-based technologies to create highly interactive platforms via which individuals and communities share, co-create, discuss, and modify user-generated content” ([2], p. 1). Adolescents are particularly active users, with nearly half saying that they use social media “almost constantly” [3]. Among Norwegian 16–18-year-olds, nearly 100% are on social media [4], and 63% of boys and 84% of girls in senior high school spend a minimum of one hour on social media every day [5]. 

There is growing literature on the potential consequences of adolescents’ social media use [6]. Overall, meta-analyses point to a small negative effect of social media use on adolescents’ mental health and well-being [7,8,9]. However, most of these studies have focused primarily on the duration and frequency of social media use [6,7,10] and provide little insight into how specific types of social media use may be differentially related to mental health and well-being [7]. Some studies have, however, demonstrated that the associations between social media use and mental health depend on the type of use [11,12,13,14,15], the motivations for use [16], and the emotional investment in social media [17,18,19,20]. For example, some studies have indicated that passive use (e.g., scrolling through others’ content) leads to a decline in well-being, while active use (e.g., interacting with people on social media) improves or has no effect on well-being [21,22]. A recent study, however, showed that only 10% of their adolescent participants felt worse after passively using social media, while 46% felt better and 44% experienced no change in well-being, demonstrating the importance of person-specific effects of social media use [23]. Exploring differences in adolescents’ social media use may bring us closer to determining whether specific types of social media use are harmful and who might be at risk of experiencing negative effects of their social media use.

Self-presentation and social comparison on social media have gained research attention for their potential effects on mental health [7,13,16,24,25,26,27,28,29]. The influential dual-factor model proposed by Nadkarni & Hofman [30] posits that, alongside the need for belonging, the need for self-presentation is a fundamental motivation for using social media. Self-presentation is the innate tendency of attempting to manage how other people perceive us [31] and entails putting up a desired image of oneself with the hope of gaining positive feedback and social approval from others [32,33]. As described in the hyperpersonal model [34,35], social media and other computer-mediated communication allow people to conceal undesirable characteristics and highlight desirable characteristics to a larger degree than in face-to-face communication—for example, by optimizing messages before sending or posting them or by carefully selecting and editing photos of oneself. In addition to providing more opportunities for self-presentation, social media also offer a range of opportunities for feedback on one’s self-presentation. On social media, feedback often comes in the form of likes, comments, and other indicators of approval or disapproval, such as having your content shared by others (e.g., “retweets”) or losing followers on your social media account. While face-to-face feedback is often ambiguous and open to interpretation, feedback on social media often represents quantifiable indicators of one’s social success [36], which can be directly compared to others’ success. In order to elicit a more favorable response, people may engage in strategic self-presentation, such as editing photos or deleting content that does not receive the desired number of likes [37]. Self-presentation on social media, which is motivated by getting positive feedback, referred to as feedback-seeking or status-seeking, has been associated with negative outcomes such as depressive symptoms [25], lower body satisfaction, and lower well-being [16]. Feedback-seeking may also influence some people to present themselves on social media in a way that does not correspond with their personality or physical appearance offline. Inauthentic self-presentation on social media has been associated with elevated levels of social anxiety and lower self-esteem [27]. 

Although not necessarily resorting to inauthentic self-presentation, people tend to emphasize desirable characteristics on social media [38], resulting in social media being dominated by idealized and unrealistic presentations of peoples’ lives (and looks). Consequently, social media are fertile grounds for upward social comparison. Social comparison is the tendency to compare one’s abilities and opinions to other people to gain information about how we are doing relative to others [39]. Upward social comparison happens when one compares oneself to someone who is viewed as better in some respect, which may be particularly prevalent on social media. One study found that social media users generally assumed that other users have more friends, are happier, and have better lives than themselves [40]. Furthermore, by following a large number of people on social media, the reference group to which adolescents compare themselves may include a very large number of people and even high-status celebrities and “influencers” [41]. Upward social comparison has been associated with more depressive symptoms [25,42] and body dissatisfaction among adolescents [28] and with suicidal ideation among young adults [13,29]. One recent study found that increased levels of feedback-seeking and social comparison were associated with more depressive symptoms, anxiety, and reduced well-being among adolescents [43]. 

In summary, aspects of self-presentation on social media such as feedback-seeking, strategic self-presentation, and social comparison may represent risk factors for experiencing negative effects of social media use. Importantly, self-presentation on social media is not inherently negative; it has been shown to have positive effects under certain conditions. Experimental studies have shown that viewing one’s own social media profile, which often portrays oneself in a positive way, leads to increased self-esteem [44,45] and an improved ability to cope with negative feedback [46]. This may be seen in connection with self-affirmation theory, which posits that a threat to people’s image of themselves as a “good or lovable person” results in them trying to restore their self-integrity [47,48]. As such, posting positive content about oneself on social media may be a way to try to restore a sense of worth. In order to result in self-affirmation, however, the content needs to be accurate [46]. Another potential benefit of social media is when they enable the expression of aspects of the self that are perceived as unwanted in offline social settings, thus allowing people to engage in a more authentic self-presentation online than offline [49].

Given the associations of feedback-seeking, strategic self-presentation, and upward social comparison with mental health and well-being, individual differences in upwards social comparison and aspects of self-presentation may underlie some of the heterogeneity in the effects of social media use on well-being. Previous studies have pointed to some characteristics that are related to self-presentation and social comparison on social media. Firstly, adolescent girls have been found to report higher levels of feedback-seeking and social comparison than boys [25,43]. They have also been shown to post more self-focused images (“selfies”) than adolescent boys, to be more focused on their physical appearance, and to be more concerned about peer-feedback [50]. Additionally, some studies suggest that personality is associated with aspects of self-presentation on social media. According to the Five Factor Model of personality, personality characteristics cluster into five traits that predict people’s behavior in a wide range of contexts [51,52]: agreeableness, conscientiousness, extraversion, neuroticism, and openness to experience. Agreeableness is associated with greater concern regarding getting along with other people, and conscientiousness is associated with striving for achievement and self-discipline [53]. Both imply that they would be cautious in how they present themselves online [54], as the opposite could result in hurting their likeability and their status. Conscientiousness and agreeableness have been negatively associated with attention-seeking [54]. Extraversion is associated with seeking social attention [55], and it has been shown that extroverts place importance on looking popular on social media [56]. Neuroticism is associated with low emotional stability, and being high on this trait means you are more susceptible to negative emotions [53]. People with high neuroticism are more sensitive to rejection, which may lead them to seek acceptance through social media [54]. Neuroticism is also associated with the tendency to present an idealized or inauthentic version of oneself [27]. Finally, openness to experience is associated with open-mindedness, novelty-seeking, and curiosity [52]. Openness has been associated with overall social media use [57], but it is unclear whether it is associated with self-presentation. 

Aspects of self-presentation on social media have also been related to lifestyle variables. Nesi & Prinstein [37] found that feedback-seeking on social media was associated with substance abuse and risky sexual behavior, and they hypothesized that those high in feedback-seeking engage in risky offline behaviors that are considered popular among peers in an attempt to increase their social status [37]. Other lifestyle factors such as physical exercise have not been investigated specifically in relation to aspects of self-presentation, but higher social media use in general is associated with higher sedentary time among adolescents [58,59]. Problematic social media use, measured using addiction criteria, has been associated with lower levels of physical activity among girls and with higher levels of physical activity among boys [60,61].

Increased knowledge about individual differences in self-presentation on social media and how these differences relate to other aspects of adolescents’ lives may help identify those at risk of negative effects of their social media use. The aim of the present study was to explore how adolescents differ in upward social comparison and aspects of self-presentation and to assess whether such differences were associated with sociodemographic variables, lifestyle, and personality.

## 2. Materials and Methods

### 2.1. Study Design and Setting

The present study (OSF preregistration doi: 10.17605/OSF.IO/BVPS8) was based on cross-sectional data from the “LifeOnSoMe” study, an online survey conducted in Bergen, Norway in 2020. Bergen is the second-largest city and a municipality in Vestland county in Western Norway, with a population of around 300,000. All senior high school pupils in the municipality of Bergen aged 16 years or older were invited to complete the survey. The survey was completed during school hours in collaboration with school personnel. Pupils from 12 schools participated, while 2 schools did not have the capacity to prioritize the survey and declined participation. Thus, a total of 3959 pupils were invited to participate in the survey, of which 2116 agreed to participate (54%). Those with missing data on gender and/or age were excluded from the analyses (n = 71), and those reporting non-binary gender were excluded due to privacy concerns (n = 13). Upon analysis, nine responses were excluded, as they were duplicates (i.e., they completed the survey twice), leaving a total number of respondents of 2023.

### 2.2. Variables

#### 2.2.1. Social Media Use: Background Information

To assess the participants’ frequency of social media use, we asked them the following question: “How often do you use social media?” The response alternatives were “almost never”, “several times a month, but rarer than once a week”, “1–2 times per week”, “3–4 times per week”, “5–6 times per week”, “every day”, “several times each day”, and “almost constantly”. For the purpose of the present study, we differentiated between “daily or less” (21%), “many times a day” (51%), and “almost constantly” (28%). To assess participants’ duration of social media use, we asked the following question: “On the days that you use social media, approximately how much time do you spend on social media?” There were seven response alternatives ranging from “less than 30 min” to “more than 5 h”. For the purpose of this study, we differentiated between “<2 h” (28%), “2–4 h” (36%), “4–5 h” (21%), and “>5 h” (14%).

#### 2.2.2. Self-Presentation and Upward Social Comparison Inclination Scale (SPAUSCIS)

The items used to assess upward social comparison and aspects of self-presentation were developed based on focus group interviews with senior high school pupils [62]. The topic of the focus group interviews was the role of social media in relation to adolescents’ mental health and well-being. Based on the focus group interviews, a new battery of questions related to different aspects of social media use covering 13 topics was developed [62]. To assess the relevance, wording, and content validity of the included items, a resource group consisting of adolescents (n = 7, age range 16–19) tested the questionnaire, and questions were revised based on their feedback. The final questionnaire was piloted in a senior high school outside of Bergen in 2020 (n = 513) [43]. In the present study, the items potentially most relevant to upward social comparison and self-presentation were used and collectively called the “Self-presentation and Upward Social Comparison Inclination Scale” (SPAUSCIS). The development of the items is also described elsewhere [43]. The items included in the SPAUSCIS were:

1. I use a lot of time and energy on the content I post on social media
2. It is important to me that my posts receive many likes and/or comments
3. It is important to me to have many followers on social media
4. I delete posts on social media that do not receive enough likes and/or comments
5. I retouch images of myself to look better before I post them on social media
6. It’s easier to be myself on social media
7. What others post on social media (images/status updates/stories) makes me feel less content with myself and my life
8. The response I get for what I post (images/status updates/stories) impacts how I feel
9. I don’t care about how many likes or comments I receive on social media

The response categories were “not at all”, “very little”, “sometimes/partly true”, “a lot”, and “very much”, coded 1–5.

#### 2.2.3. Sociodemographic and Background Variables

The participants reported their age and gender, which education program they attended, and their country of birth. In Norway, pupils can choose a study preparation program to achieve a general university admissions certification or a vocational education program leading to vocational competence in skilled trades. Subjective socioeconomic status (SES) was assessed using the following question: “How well off do you consider your own family to be compared to others?” The response categories ranged from 0 (“Very poor”) to 10 (“Very well off”). For this study, age was recoded so that all participants of 18 years of age or more (max 21) were combined into one group (18+). SES was recoded to a tripartite variable of low SES (scores 0–4; 6.2%), medium SES (5–7, 51.3%), and high SES (8–10, 42.4%).

#### 2.2.4. Lifestyle Factors

The participants indicated how often they exercised each week, following this description of exercise: “By exercise we mean that you go for a walk, go skiing, swimming, or other exercise activities/sports”. The response alternatives were: “Never”, “less than once a week”, “once a week”, “2–3 times a week”, “4–6 times a week”, and “about every day”. The variable was dichotomized to “low/moderate exercise” (2–3 times a week or less; 57%) and “high exercise” (4–6 times a week or more; 43%). 

The participants were asked how often they drank alcohol, smoked cigarettes, and used snus (under-lip smoke-less tobacco). The participants indicated how often they normally drink over a two-week period, and a variable with four levels was created: never tried alcohol (“Never”; 24%), less often than once in two weeks (“Rarely”; 28%), one to two times in two weeks (“Regularly”; 41%), and more often than two times in two weeks (“Often”; 7%). For cigarettes and snus, the response alternatives were combined, and the variables dichotomized in order to compare those who had tried cigarettes (39%) or snus (36%) with those who had not (61% for cigarettes and 64% for snus).

#### 2.2.5. Personality

Personality was assessed using the Ten-Item Personality Inventory (TIPI) [63]. The TIPI measures the Big Five personality dimensions of extraversion, agreeableness, conscientiousness, emotional stability, and openness to new experiences using ten items measuring two opposing traits for each dimension. The ten items are preceded by the heading “I see myself as”, followed by trait descriptive adjectives. The response categories range from 1 (strongly disagree) to 7 (strongly agree). Each participant received a total score for each personality trait by recoding the reverse-scored items and taking the average of the two items. The Spearman Brown coefficient, which is a measure of reliability recommended for scales with two items [64], was 0.71 for extraversion, 0.30 for agreeableness, 0.55 for conscientiousness, 0.63 for emotional stability, and 0.34 for openness to experience. For the purpose of this study, we created tripartite variables for each personality trait denoting a low (1st–33rd percentile), moderate (34th–66th percentile), and high (67th–100th percentile) score on each trait. In our sample, the proportion scoring low, moderate, and high was 32%, 35%, and 33% for extraversion, 25%, 33%, and 41% for agreeableness, 29%, 38%, and 33% for conscientiousness, 28%, 34%, and 37% for emotional stability, and 28%, 44%, and 28% for openness.

### 2.3. Analysis

#### 2.3.1. Structural Validity of the SPAUSCIS

Exploratory factor analysis was used to examine the number of underlying factors in the SPAUSCIS, and internal validity was assessed by Cronbach’s alpha, using the “jmv” package in R [65]. A confirmatory analysis (CFA) was performed using the “lavaan” package in R [66].

#### 2.3.2. Identifying the Number of Classes and the Description of Retained Classes

Latent class analysis (LCA) was used to identify classes of participants sharing similar response patterns on the items of the SPAUSCIS [67]. LCA is person-centered and model-based, assumes a parametric statistical model, and uses the observed data to estimate parameter values for the selected model [68]. Several statistical criteria were used to establish the most appropriate number of latent classes. For the Akaike information criterion (AIC) and Bayesian information criterion (BIC), lower values indicate a better model fit [69]. Relative entropy (range 0–1) assesses the quality of classification, where a higher value indicates better discrimination between the classes, and the Lo–Mendell–Rubin ad hoc adjusted likelihood ratio test (LMR-LR) indicates whether a given model performs better than a model with k-1 classes. The LCA was performed using the “poLCA” package in R [70], while relative entropy and LMR-LR were calculated using Mplus [71].

#### 2.3.3. Class Belongingness and Covariates

Multinominal logistic regression was used to assess the relationship between class membership and sociodemographic variables, lifestyle, and personality, and it is expressed in relative risk ratios with corresponding 95% confidence intervals. The multinominal logistic regression was performed using the “nnet” package in R [72]. The associations were estimated separately for each covariate. Gender is likely to be an important confounding variable, as it has been shown to be related to both upward social comparison and self-presentation [73,74,75] and to several of the covariates [76,77,78]. Therefore, the multinominal logistic regressions were run with and without controlling for gender.

#### 2.3.4. Missing Data

There were some missing data for the self-presentation data (from 2.8% on “followers important” to 4.9% on “I don’t care”). For the CFA, listwise deletion is the default [66]. In the LCA, cases with missing values are retained, and class membership is estimated based on the available information [70]. In all analyses of associations, pairwise deletion was used to retain as many of the data as possible.

## 3. Results

The mean age of the sample was 17.36 years (SD 0.85), and 56% were girls (Table 1).

Table 2 shows the frequency and duration of social media use in total and separately for boys and girls. There were significant differences between boys and girls in terms of frequency and duration of use. A total of 83% of the girls indicated that they used social media several times each day or “almost constantly”, compared to 74 among boys.

### 3.1. Structural Validity of the SPAUSCIS

The correlation matrix including the nine items revealed that the items “It is easier to be myself on social media” and “I don’t care about how many likes or comments I receive on social media” had no correlations with other items >0.30, suggesting that these items should be excluded. The correlation matrix is available as Supplementary Materials (Table S1). The seven remaining self-presentation items were subjected to an exploratory factor analysis (EFA), with no rotation, using principal axis factoring, as the data had a non-normal distribution. The Kaiser–Meyer–Olkin measure of sampling adequacy was 0.86, verifying the sampling adequacy of the analysis [79]. Bartlett’s test of sphericity (X^2^ (21) = 6993, *p* < 0.001) supported the factorability of the correlation matrix. The eigenvalue was 3.59 for one factor and dropped to 0.27 for two factors, strongly suggesting a unidimensional scale. With one factor, the model explained 51% of the variance. 

**Figure 1:**
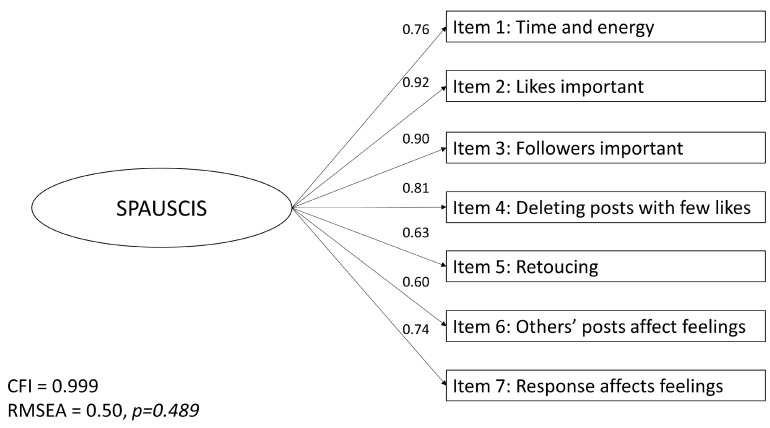
Results of the confirmatory factor analysis: One-factor model with the items “easier to be myself” and “I don’t care” deleted. CFI = Comparative Fit Index, RMSEA = root mean square error of approximation, SPAUSCIS = Self-presentation and Upward Social Comparison Inclination Scale.

A CFA was completed with the seven retained items. Items 2 (likes important) and 3 (followers important) and items 6 (others posts impact feelings) and 7 (response impacts feelings) had highly correlated error terms, and these correlations were allowed in the final model. The CFA resulted in a Comparative Fit Index (CFI) of 0.999, a root mean square error of approximation (RMSEA) of 0.050 (95%CI 0.039–0.062, *p* = 0.489), and a standardized root mean square residual (SRMR) of 0.021, all signaling good fit [80]. The loadings of items 1–7 varied from 0.60 (item 6: “others’ posts affect feelings”) to 0.92 (item 2: “likes are important”), with a mean of 0.77 (Figure 1). The seven items of the SPAUSCIS tap into upward social comparison and different aspects of self-presentation, which we collectively refer to as “focus on self-presentation”.

### 3.2. Number and Characteristics of Latent Classes

In the LCA, models with 1–7 latent classes were run with 50 repetitions each and with random starting values to assess model identifiability [81]. Table 3 shows the fit for models with 1–5 latent classes. AIC, BIC, and relative entropy all improved up to three classes, after which they decreased only slightly. The LMR-LR indicated a statistically significant improvement of the model when moving from a 2-class to a 3-class model and no improvement when moving to a 4-class model. Based on these fit criteria and a visual inspection of the meaningfulness of models with 2, 3, 4, and 5 classes, a 3-class solution was chosen.

**Figure 2:**
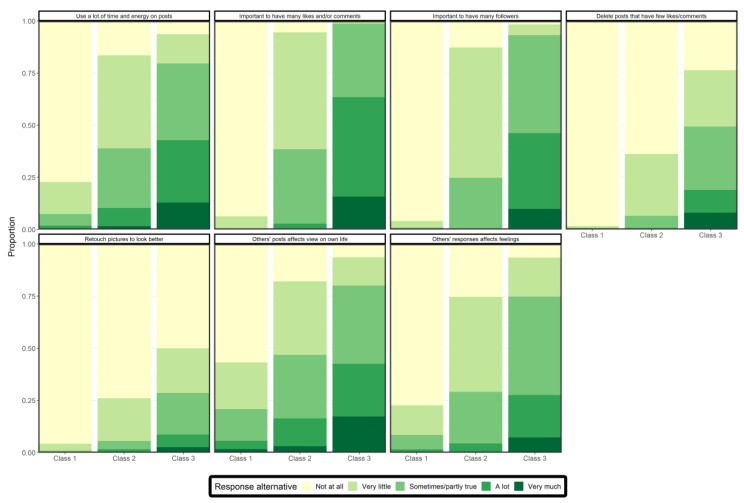
Response probabilities on the self-presentation scale across retained classes.

The classes represent response patterns across the seven items. The predicted class membership by modal posterior probability was 42% in class 1, 33% in class 2, and 25% in class 3. Figure 2 shows the distribution of most probable responses for each item in each of the three latent classes. Table 4 shows the probability of endorsing (i.e., responded “sometimes/partly true” or higher) the items for each class. In the first class, there were low probabilities of endorsing the items. The highest probabilities were found for items 1, 6, and 7 (8%, 21%, and 9%, respectively). In class 2, the probabilities of endorsing the items ranged from 5–46%, where items 1, 2, and 6 had the highest probabilities of endorsement (38%, 38%, and 46%, respectively). In class 3, there were high probabilities of endorsement of all items (29–99%). In this class, the items with the highest probabilities of endorsement were items 2 and 3 (99 and 95%), followed by item 6 (81%). Based on the conditional probability results of classes 1, 2, and 3, we named class 1 “Low focus on self-presentation”, class 2 “Intermediate focus on self-presentation”, and class 3 “High focus on self-presentation”.

### 3.3. Class Belongingness and Covariates

**Figure 3:**
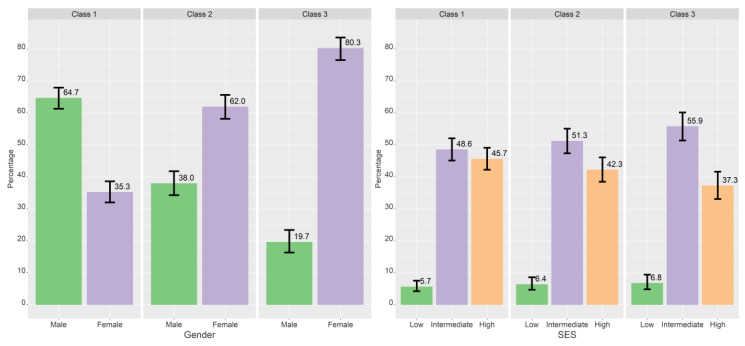
Crude proportions for each class across gender, age, and subjective socioeconomic status. Proportions based on most probable class belongingness. The error bars denote 95% confidence intervals.

**Figure 4:**
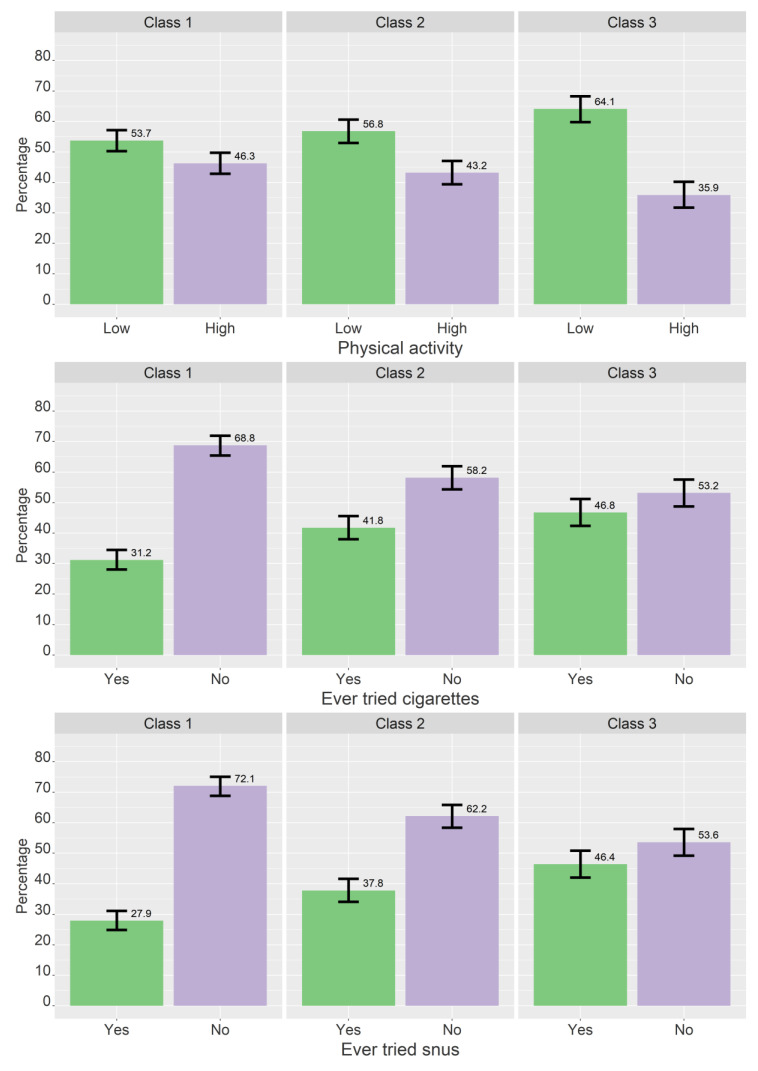
Crude proportions for each class across physical activity, cigarettes (ever tried, yes/no), and snus (ever tried, yes/no). Proportions based on most probable class belongingness. The error bars denote 95% confidence intervals.

**Figure 5:**
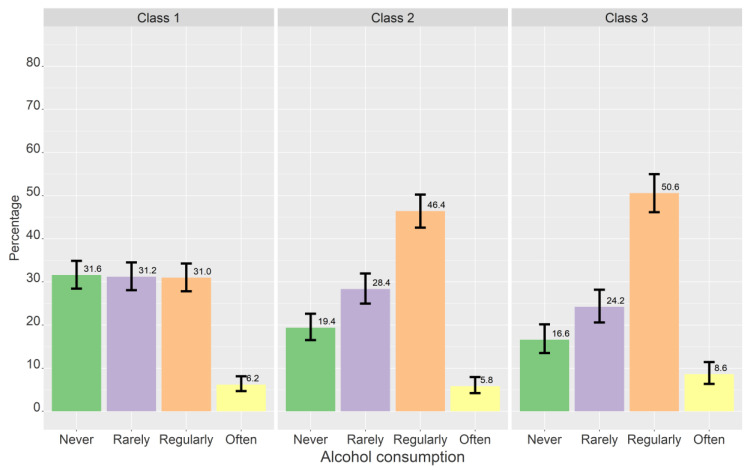
Crude proportions for each class across alcohol consumption. Proportions based on most probable class belongingness. The error bars denote 95% confidence intervals.

**Figure 6:**
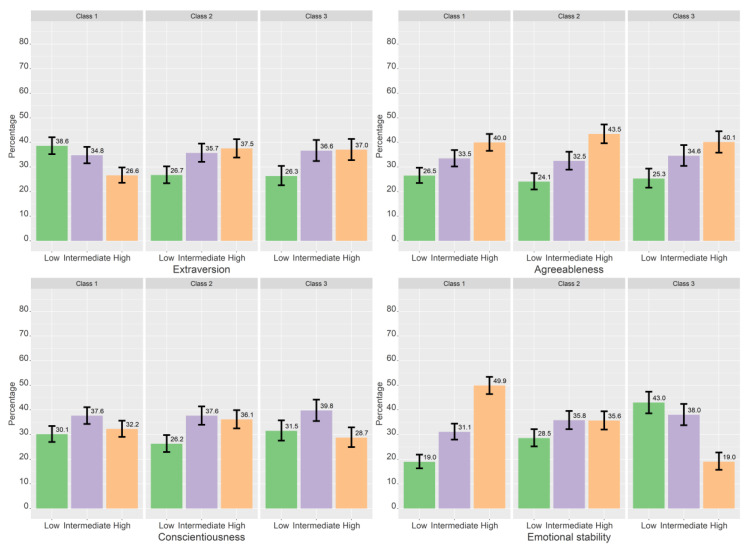
Crude proportions for each class across personality traits. Proportions based on most probable class belongingness. The error bars denote 95% confidence intervals.

Table 5, Table 6 and Table 7 shows the results of the multinominal logistic regression, both with and without controlling for gender. Figure 3, Figure 4, Figure 5 and Figure 6 show the estimated proportions across sociodemographic, lifestyle, and personality variables (not controlled for gender).

#### 3.3.1. Sociodemographic Factors

Girls were more likely to be in higher classes (i.e., a higher focus on self-presentation) compared to lower classes for all class comparisons (see Table 5). Those with intermediate SES were more likely than those with high SES to be in class 3 compared to class 1. This association became non-significant when controlling for gender. There was no statistically significant difference in age across classes.

**Table 5:** Comparison of class belongingness across sociodemographic variables.

	Class 2 vs. Class 1 (Intermediate vs. Low Focus on Self-Presentation)	Controlled for Gender	Class 3 vs. Class 1 (High vs. Low Focus on Self-Presentation)	Controlled for Gender	Class 3 vs. Class 2 (High vs. Intermediate Focus on Self-Presentation)	Controlled for Gender
	**RRR (95 CI)**	**RRR (95 CI)**	**RRR (95 CI)**	**RRR (95 CI)**	**RRR (95 CI)**	**RRR (95 CI)**
**Gender**						
Boy	Ref.	Ref.	Ref.	Ref.	Ref.	Ref.
Girl	2.99 (2.42–3.59) ****	-	7.48 (5.77–9.70) ****	-	2.50 (1.91–3.27) ****	-
**Age**						
16	0.97 (0.69–1.36)	1.04 (0.73–1.47)	0.99 (0.69–1.43)	1.11 (0.75–1.65)	1.03 (0.70–1.51)	1.08 (0.73–1.59)
17	1.01 (0.81–1.25)	1.06 (0.84–1.32)	0.96 (0.76–1.25)	1.04 (0.81–1.34)	0.96 (0.75–1.22)	0.99 (0.77–1.26)
18	Ref.	Ref.	Ref.	Ref.	Ref.	Ref.
**SES**						
Low	1.22 (0.78–1.90)	0.97 (0.61–1.53)	1.47 (0.92–2.35)	1.00 (0.61–1.66)	1.20 (0.74–1.95)	1.04 (0.64–1.69)
Intermediate	1.14 (0.92–1.41)	1.02 (0.82–1.27)	1.41 (1.12–1.77) ***	1.17 (0.92–1.50)	1.23 (0.97–1.57)	1.15 (0.90–1.46)
High (ref)	Ref.	Ref.	Ref.	Ref.	Ref.	Ref.

Note: 95 CI = 95% confidence interval; Ref = reference (base) class for comparison of two classes, RRR = relative risk ratio. *** *p* < 0.005, **** *p* < 0.001.

#### 3.3.2. Lifestyle

Compared to those who had never tried alcohol, those who consumed alcohol more than twice in two weeks were more likely to be in higher classes for all class comparisons when controlling for gender (Table 6). Those who consumed alcohol 1–2 times in two weeks were more likely to be in classes 2 and 3 compared to class 1, while those who consumed alcohol less than once in two weeks were more likely to be in class 2 compared to class 1. Those who had tried cigarettes or snus were more likely to be in higher classes for all class comparisons. Those with low/moderate physical activity were more likely to be in class 3 compared to class 1, but this association became non-significant when controlling for gender.

**Table 6:** Comparison of class belongingness across lifestyle variables.

	Class 2 vs. Class 1 (Intermediate vs. Low Focus on Self-Presentation)	Controlled for Gender	Class 3 vs. Class 1 (High vs. Low Focus on Self-Presentation)	Controlled for Gender	Class 3 vs. Class 2 (High vs. Intermediate Focus on Self-Presentation)	Controlled for Gender
**Physical activity**						
Low/moderate	1.13 (0.92–1.39)	0.96 (0.77–1.18)	1.54 (1.23–1.93) ****	1.15 (0.90–1.47)	1.36 (1.07–1.72) *	1.21 (0.95–1.54)
High	Ref.	Ref.	Ref.	Ref.	Ref.	Ref.
**Alcohol**						
Never	Ref.	Ref.	Ref.	Ref.	Ref.	Ref.
Rarely	1.48 (1.12–1.96) **	1.43 (1.09–1.91) *	1.48 (1.07–2.04) *	1.39 (0.98–1.96)	1.00 (0.70–1.42)	0.97 (0.68–1.39)
Regularly	2.44 (1.87–3.19) ****	2.25 (1.71–2.96) ****	3.11 (2.30–4.19) ****	2.70 (1.96–3.72) ****	1.27 (0.93–1.75)	1.20 (0.87–1.66)
Often	1.53 (0.96–2.44)	1.71 (1.06–2.76) *	2.64 (1.65–4.23) ****	3.25 (1.95–5.42) ****	1.73 (1.04–2.87) *	1.90 (1.13–3.19) *
**Ever tried cigarettes**						
No	Ref.	Ref.	Ref.	Ref.	Ref.	Ref.
Yes	1.58 (1.28–1.96) ****	1.77 (1.42–2.21) ****	1.94 (1.54–2.43) ****	2.34 (1.83–3.00) ****	1.22 (0.97–1.54)	1.32 (1.04–1.67) *
**Ever tried snus**						
No	Ref.	Ref.	Ref.	Ref.	Ref.	Ref.
Yes	1.57 (1.26–1.95) ****	1.75 (1.40–2.19) ****	2.24 (1.77–2.82) ****	2.69 (2.09–3.46) ****	1.42 (1.13–1.80) ***	1.54 (1.21–1.95) ****

Note: 95 CI = 95% confidence interval; Ref = reference (base) class for comparison of two classes, RRR = relative risk ratio. * *p* < 0.05. ** *p* < 0.01, *** *p* < 0.005, **** *p* < 0.001.

#### 3.3.3. Personality

Compared to low extraversion, those with intermediate or high extraversion were more likely to be in classes 2 and 3 compared to class 1 (Table 7). When controlling for gender, those with high agreeableness (vs. low agreeableness) had a lower likelihood of belonging to class 3 compared to class 1. For conscientiousness, those with high scores had a lower likelihood of being in class 3 compared to class 1 (when controlling for gender) and class 2. Compared to high emotional stability, those with low or intermediate emotional stability were more likely to be in higher classes compared to lower classes for all class comparisons. When controlling for gender, the increased likelihood of being in class 3 vs. class 2 for those with intermediate emotional stability became non-significant. There were no statistically significant differences for agreeableness across classes.

**Table 7:** Comparison of class belongingness across personality variables.

	Class 2 vs. Class 1 (Intermediate vs. Low Focus on Self-Presentation)	Controlled for Gender	Class 3 vs. Class 1 (High vs. Low Focus on Self-Presentation)	Controlled for Gender	Class 3 vs. Class 2 (High vs. Intermediate Focus on Self-Presentation)	Controlled for Gender
**Extraversion**						
Low	Ref.	Ref.	Ref.	Ref.	Ref.	Ref.
Intermediate	1.49 (1.15–1.91) ***	1.58 (1.22–2.05) ****	1.54 (1.17–2.03) ***	1.71 (1.28–2.31) ****	0.92 (0.68–1.25)	0.96 (0.70–1.31)
High	2.04 (1.57–265) ****	2.16 (1.65–2.83) ****	2.04 (1.54–2.71) ****	2.25 (1.66–3.05) ****	0.95 (0.73–1.23)	0.98 (0.75–1.28)
**Agreeableness**						
Low	Ref.	Ref.	Ref.	Ref.	Ref.	Ref.
Intermediate	1.07 (0.81–1.40)	0.95 (0.71–1.25)	1.08 (0.81–1.45)	0.87 (0.63–1.19)	1.02 (0.78–1.34)	0.97 (0.73–1.27)
High	1.20 (0.92–1.55)	0.97 (0.74–1.27)	1.05 (0.79–1.39)	0.73 (0.53–0.99) *	0.90 (0.67–1.20)	0.78 (0.58–1.05)
**Conscientiousness**						
Low	Ref.	Ref.	Ref.	Ref.	Ref.	Ref.
Intermediate	1.15 (0.89–1.48)	1.09 (0.84–1.42)	1.01 (0.77–1.32)	0.92 (0.69–1.23)	0.91 (0.68–1.21)	0.89 (0.66–1.19)
High	1.29 (0.99–1.67)	1.15 (0.88–1.51)	0.85 (0.64–1.13)	0.71 (0.52–0.96)	0.69 (0.52–0.90) **	0.64 (0.49–0.85) ***
**Emotional stability**						
Low	2.11 (1.61–2.75) ****	1.45 (1.09–1.93) *	5.95 (4.39–8.06) ****	3.24 (2.34–4.49) ****	2.85 (2.03–4.02) ****	2.20 (1.54–3.14) ****
Intermediate	1.61 (1.27–2.05) ****	1.29 (1.00–1.65) *	3.21 (2.40–4.30) ****	2.19 (1.61–2.98) ****	1.70 (1.18–2.46) ***	1.45 (1.00–2.10)
High	Ref.	Ref.	Ref.	Ref.	Ref.	Ref.
**Openness**						
Low	Ref.	Ref.	Ref.	Ref.	Ref.	Ref.
Intermediate	1.15 (0.89–1.48)	1.15 (0.88–1.49)	0.87 (0.67–1.14)	0.87 (0.65–1.16)	0.98 (0.74–1.29)	0.97 (0.73–1.28)
High	1.19 (0.90–1.58)	1.28 (0.96–1.71)	0.91 (0.68–1.23)	1.03 (0.75–1.42)	0.90 (0.68–1.19)	0.94 (0.71–1.25)

Note: 95 CI = 95% confidence interval; Ref = reference (base) class for comparison of two classes, RRR = relative risk ratio. * *p* < 0.05. ** *p* < 0.01, *** *p* < 0.005, **** *p* < 0.001.

## 4. Discussion

In this exploratory study, we assessed differences among adolescents’ upward social comparison and aspects of self-presentation on social media and whether these differences were related to gender, age, SES, lifestyle factors, or personality. Over 2000 Norwegian senior high school pupils participated in the study. The results showed that feedback-seeking, strategic self-presentation, and social comparison on social media could be combined into one factor, referred to here as “focus on self-presentation”. The experience of it being easier to be oneself on social media did not correlate with the other aspects of self-presentation measured in this study and was excluded from the SPAUSCIS. It is possible that this item taps into other aspects of social media use. For example, some people may be less shy and withdrawn online than offline [82,83,84], or aspects of the self that are hidden or suppressed in offline interactions can be more freely expressed on social media [85]. 

The latent class analysis identified three groups of adolescents who varied in their focus on self-presentation. We named the classes “low focus on self-presentation” (class 1), “some focus on self-presentation” (class 2), and “high focus on self-presentation” (class 3). Group membership was associated with gender, lifestyle factors, and personality traits, where being a girl, having intermediate and high extraversion, having low and intermediate emotional stability, consuming alcohol, and having tried cigarettes and snus increased the likelihood of a higher focus on self-presentation. There was some indication that those with high agreeableness and high conscientiousness were less likely to have a high focus on self-presentation. The associations between focus on self-presentation and SES and between focus on self-presentation and low/moderate physical activity both became non-significant when controlling for gender. Focus on self-presentation was not related to age or the personality trait of openness to new experiences.

Using the same items as those in the present study, Skogen et al. [43] found a higher score on the items among adolescent girls than boys. Our results corroborate these findings, using a larger and more heterogeneous sample (pupils from twelve schools in rural and central areas). Class 1 (low focus on self-presentation) was dominated by boys, while class 2 (intermediate focus on self-presentation) and class 3 (high focus on self-presentation) were characterized by successively larger estimated proportions of girls. On a similar note, studies have shown that females both post selfies and retouch selfies before posting them to a greater degree than males [12,86]. The higher proportion of girls in class 2 and 3 can be understood in the context of the stronger tendency among adolescent females to have a relational orientation and increased reactivity to interpersonal stressors compared to males [87,88]. Some studies suggest that the association between social media use and negative mental health outcomes is stronger among females [20,89]. The increased focus on self-presentation may be one contributing factor to this relationship. 

There was some evidence for a relationship between SES and group membership, with an increased relative risk of having a high focus on self-presentation for those with intermediate as compared to high SES. This relationship, however, became non-significant when controlling for gender. To the authors’ knowledge, no other studies have assessed the relationship between self-presentation and SES, but SES has been related to other aspects of social media use and, more generally, to screen use. For instance, low SES has been associated with social media addiction among children and adolescents [90], and access to media devices in the bedroom is more common among adolescents from low-income families compared to high-income families [91]. Overall, our sample was characterized by relatively high SES, and studies on more diverse populations should be conducted to better illuminate the relationship between focus on self-presentation and SES. 

The lack of an association between age and group membership may be due to the limited age range of the participants in this study. Social media use is common from a young age, and among Norwegian children, one-fourth of boys and one-third of girls already use Snapchat at the age of 9–10 years [4]. Adolescents’ online self-presentation has been shown to change with age [92] and to be influenced by identity development [93]. Among 13–18-year-olds, Fullwood et al. [92] showed that younger adolescents were more likely than older adolescents to present an idealized or false version of themselves online and to experiment with multiple self-presentations. Among emerging adults, Michikyan [93] found that those high in identity confusion were less realistic, less truthful, and more socially desirable in their self-presentation online than those high in identity coherence.

We found that the personality traits extraversion and emotional stability were associated with class membership. Those with high extraversion were more likely to have a higher focus on self-presentation than those with low extraversion. These findings correspond to the findings of Zywica & Danowski [56], who found that a larger proportion of extraverts relative to introverts reported that it was important to look popular on Facebook. Associations between extraversion and other aspects of social media use may also be related to the present findings. For instance, meta-analytic evidence has shown that extraversion is positively associated with the amount of social media use [94], the number of friends on social media [95], and using social media for social interaction [96]. One may speculate that extraverts use social media to fulfil their social needs and that they consequently consider social media as an important part of their social lives and become more focused on how they appear online compared to introverts. 

Emotional stability was even more strongly associated with class membership than extraversion, where the estimated proportions of low, intermediate, and high emotional stability shifted substantially with an increasing focus on self-presentation. The proportion of intermediate and low emotional stability increased with a higher focus on self-presentation, and high emotional stability decreased. This can be seen in the context of the results of Twomey & O’Reilly [27], who showed that neuroticism (i.e., low emotional stability) was associated with individuals’ tendency to present an idealized or inauthentic version of themselves online. Neuroticism has also been associated with posting more status updates [97]. More generally, emotional stability is negatively associated with the amount of social media use [94,96]. 

For agreeableness and conscientiousness, those with high scores were somewhat less likely to have a high focus on self-presentation. This is in line with a study of undergraduate students, where agreeableness and conscientiousness were associated with a lower likelihood of using social media to seek attention from others [54]. There was no relationship between class membership and openness to new experiences. High scores on openness have been associated with more social media use in studies of adults [57,96], but as social media use is ubiquitous among adolescents, this personality trait may be a more important predictor of social media use among older people [57]. 

Finally, our results show that those who consumed alcohol more frequently and those who had tried smoking and snus had increased probabilities of having an intermediate or high focus on self-presentation. This finding mirrors the findings of Nesi & Prinstein [37], who demonstrated that digital status seeking (i.e., efforts to obtain likes and comments) was longitudinally associated with substance use. The authors of that study hypothesized that digital status seekers are at risk of engaging in risky offline behaviors that are considered popular among peers in an attempt to increase their social status [37]. For physical activity, there were increased probabilities of a high focus on self-presentation for those with low/moderate physical activity compared to those with high physical activity, although not when controlling for gender. To our knowledge, no studies have looked specifically at self-presentation on social media and physical activity; however, studies have shown that low physical activity is associated with smartphone addiction [60,61] and, more generally, with high overall screen times [58,59]. 

### 4.1. Implications

Grouping adolescents by their focus on self-presentation may be one way to bring structure to the heterogeneity of adolescents’ social media use, but further work is needed to assess whether the three-class solution in the present study is relevant in other populations. Further work is also needed to assess how focus on self-presentation is related to important adolescence outcomes such as mental health, satisfaction with life, and educational attainment. Importantly, social media use is likely to differ in other areas in addition to self-presentation; however, focus on self-presentation seems to be a meaningful dimension that warrants further study. The present results can help identify groups of adolescents that are of risk of experiencing negative effects of their social media use. Our results suggest that, among adolescents, being a girl, high extraversion, and low emotional stability are associated with an increased risk of being highly focused on self-presentation. Public health interventions promoting healthy social media use could target these groups in particular. Furthermore, it has been hypothesized that the act of self-presenting on social media, such as posting selfies, triggers an increased dependence on social approval in the form of likes and comments [98]. Thus, it is possible that efforts to reduce self-presentation behavior on social media could potentially reduce someone’s dependence on social approval. However, positive self-presentation, defined as showing positive aspects of the self online, has been shown to increase subjective well-being, possibly because it supports a positive self-image [99] and self-affirmation [46]. Thus, the relationship between the act of self-presenting on social media, one’s focus on self-presentation, and well-being is complex and needs further investigation. 

The present study did not consider how focus on self-presentation may vary across different social media platforms. For example, self-presentation on social media can vary depending on the perceived target audience [100,101]. In a qualitative study by Taber & Whittaker [101], university students explained that they were more authentic and less socially desirable on social media accounts where only their close friends could access their content. Furthermore, how one self-presents on social media can be influenced by the level of anonymity, the durability of the content (e.g., ephemeral vs. permanent content; [102]), and the visibility of the content [100]. It is unclear whether one’s focus on self-presentation, beyond how one self-presents, varies across platforms, but it is likely that some social media platforms augment users’ focus on self-presentation—for example, platforms with visual content and feedback from others as central features. Thus, it is possible that some of the gender differences in focus on self-presentation are based on gender differences in platform preference, above and beyond any differences in focus on self-presentation between boys and girls in the first place.

### 4.2. Strengths and Limitations

A strength of the present study is the use of survey items developed based on focus interviews with the target group, increasing the likelihood that the items were relevant to the participants. The data collection is recent, and the study included a large number of participants, allowing for a meaningful investigation of the focus on self-presentation on social media and its covariates. 

The study also has some important limitations. First, the items measuring focus on self-presentation are not part of an established scale. However, a pilot study using the same items showed the same factor structure and supported a unidimensional scale, and the sum score was associated with mental health and well-being [43]. The same study also showed that a higher proportion of those with a high score on the scale used highly visual social media, such as Snapchat and Instagram, compared to those with low scores [43]. Second, the reliability was low for some of the TIPI scales, specifically for agreeableness and openness to experience, and the results should be interpreted with this in mind. TIPI has, however, shown good convergence with multi-item personality inventories and good test-retest reliability [63]. Further, the study is cross-sectional, which means that we are unable to draw conclusions about cause-and-effect. Furthermore, the participant rate was somewhat low (54%). It is possible that those highly invested in social media completed the survey to a larger extent than those not invested in social media, thus causing a bias in the results. Hence, the estimated proportions of the latent classes should be interpreted with caution. However, associations are less vulnerable to bias caused by low participation rates than prevalence [103], and the associations between class membership and covariates may be considered valid despite a relatively low participation rate. 

As participants were recruited through their school, adolescents not attending school did not have the opportunity to participate in the study. However, the rate of school attendance among Norwegian adolescents is very high, with 94% of 16–18-year-olds attending senior high school [104]. The participants were drawn from a limited geographical area, and the results may not be generalizable to other countries or cultures. For example, Kolesnyk et al. [105] found that the deceptive self-presentation of physical attractiveness (e.g., retouching images to increase attractiveness) was lower in countries with more gender equality.

Only one of the self-presentation items asked explicitly about visual self-presentation, specifically about the retouching of photos to look better. Self-presentation may entail photos of oneself but also photos of friends or activities, sharing music and movies, posting opinions, etc. Future studies should consider if self-presentation through posting photos of oneself differs from other forms of self-presentation—for example, due to links with appearance-related concerns [106,107,108]. Furthermore, we used the word “retouching”, which may not fully reflect the range of ways adolescents edit their photos. For example, built-in image filters on applications such as Snapchat are frequently used by adolescents but may not have been captured by the question about retouching. Retouching may have been interpreted as more elaborate and advanced photo-editing. Future studies are likely to benefit from combining quantitative findings with qualitative data to obtain a fuller picture of adolescents’ focus on self-presentation on social media. Lastly, the present study did not distinguish between different social media platforms. This is a limitation, as some items, such as the importance of likes and comments, are not relevant to all platforms. Future studies could explore whether focus on self-presentation differs across social media platforms. 

## 5. Conclusions

In this exploratory study, we showed that feedback-seeking, strategic self-presentation, and social comparison on social media converged into one factor, referred to here as “focus on self-presentation”. Using a data-driven approach, we identified three groups of adolescents with a low, intermediate, and high focus on self-presentation. Being a girl, higher extraversion, lower emotional stability, more frequent alcohol consumption, and having tried tobacco were associated with membership in the high-focus group. There was some indication that those with high agreeableness and high conscientiousness were less likely to have a high focus on self-presentation, while SES and physical activity were associated with focus on self-presentation in crude models but not after controlling for gender. Importantly, the current study included a rather homogenous sample in terms of SES, and the relationship between focus on self-presentation and SES should be further investigated in more diverse populations. Further work is also needed to assess how focus on self-presentation is related to important adolescence outcomes such as mental health, satisfaction with life, and educational attainment. However, given the association of aspects of self-presentation with negative mental health outcomes shown in previous research, efforts to reduce the focus on self-presentation could be warranted. The present results suggest some characteristics that are associated with a higher focus on self-presentation and that could inform targeted interventions. The high focus on self-presentation found among girls could explain previous findings of stronger associations between social media use and negative mental health outcomes among girls compared to boys. Importantly, specific social media affordances and the act of self-presentation may augment one’s focus on self-presentation, and gender differences in terms of focus on self-presentation may be partly related to differences in platform preference among girls and boys. The nature of these complex associations warrants further investigation, and efforts should be made to combine qualitative and quantitative approaches.

## Figures and Tables

**Table 1:** Sociodemographic and background variables across gender. The *p*-values refer to differences between boys and girls.

	Boys (N = 899, 44%)	Girls (N = 1124, 56%)	Total (N = 2023)	*p*-Value
**Age** *				
Mean (SD)	17.32 (0.85)	17.38 (0.85)	17.36 (0.85)	0.133
**Study year** ^§^				
1	16 (1.8%)	8 (0.7%)	24 (1.2%)	0.005
2	493 (55.2%)	566 (50.5%)	1059 (52.9%)	
3	384 (43.0%)	547 (48.8%)	931 (45.2%)	
**Type of study** ^§^				
Study preparation	674 (75.2%)	966 (86.0%)	1640 (81.2%)	<0.001
Vocational	222 (24.8%)	157 (14.0%)	379 (18.8%)	
**Country of birth** ^§^				
Norway	819 (91.3%)	1005 (89.4%)	1824 (90.2%)	0.154
Other country	78 (8.7%)	119 (10.6%)	197 (9.8%)	
**Self-reported SES**				
Mean (SD)	7.43 (1.76)	6.98 (1.75)	7.18 (1.77)	<0.001

Note: SES = socioeconomic status, range 0–10. * Linear model ANOVA. ^§^ Pearson’s Chi square test.

**Table 2:** Frequency and duration of social media use across gender. The *p*-values refer to differences between boys and girls.

	Boys (N = 899)	Girls (N = 1124)	Total (N = 2023)	*p*-Value
**Frequency of use**				
Daily or less	226 (25.51%)	190 (16.95%)	416 (20.73%)	<0.001
Many times a day	439 (49.55%)	582 (51.92%)	1021 (50.87%)	
Almost constantly	221 (24.94%)	349 (31.13%)	570 (28.40%)	
**Duration of use**				
<2 h	320 (36.32%)	246 (22.02%)	566 (28.33%)	<0.001
2–4 h	326 (37.00%)	402 (35.99%)	728 (36.44%)	
4–5 h	134 (15.21%)	284 (25.43%)	418 (20.92%)	
>5 h	101 (11.46%)	185 (16.56%)	286 (14.31%)	

Note. Differences between groups assessed using Pearson’s Chi square test.

**Table 3:** AIC, BIC, relative entropy, and LMR-LR for 1–6 classes in the latent class analysis.

Number of Classes	AIC	BIC	Relative Entropy	LMR-LR
1	34,358.99	34,526.14	-	-
2	29,549.39	29,914.30	0.904	*p* < 0.001
*3*	*28,306.27*	*28,788.93*	*0.879*	*p < 0.001*
4	27,902.47	28,547.89	0.878	*p* < 0.759
5	27,687.25	28,495.43	0.877	*p* < 0.759

Note. Data in italics indicate the best fitting model relative to the other models tested. AIC = Akaike information criterion; BIC = Bayesian information criterion; LMR-LR = Lo–Mendell–Rubin ad hoc adjusted likelihood ratio test.

**Table 4:** The probability of endorsing (i.e., responding “sometimes/partly true”, “a lot”, or “very much”) each of the SPAUSCIS items across retained classes.

	Class 1 (n = 839, 42%)	Class 2 (n = 671; 33%)	Class 3 (n = 513; 25%)
I use a lot of time and energy on the content I post on social media	7.5%	38.3%	80.4%
2. It is important to me that my posts receive many likes and/or comments	<1.0%	38.7%	98.8%
3. It is important to me to have many followers on social media	<1.0%	24.0%	94.5%
4. I delete posts on social media that do not receive enough likes and/or comments	<1.0%	5.7%	50.4%
5. I retouch images of myself to look better before I post them on social media	<1.0%	5.1%	29.3%
6. What others post on social media (images/status updates/stories) makes me feel less content with myself and my life	20.9%	46.0%	81.1%
7. The response I get for what I post (images/status updates/stories)	8.6%	27.7%	76.5%

## Data Availability

The datasets analyzed during the current study are not publicly available, as they contain sensitive information, and the ethical approval of the study does not include this option. The datasets will be available from the corresponding author on reasonable request.

## Supplementary Materials

The following supporting information can be downloaded at: https://www.mdpi.com/article/10.3390/ijerph191711133/s1, Table S1: The correlation matrix (non-parametric) of candidate self-presentation items.
