# Supplementary material for: Focus on Self-Presentation on Social Media across Sociodemographic Variables, Lifestyles, and Personalities: A Cross-Sectional Study
Source: Int J Environ Res Public Health. 2022 Sep 5;19(17):11133. doi: 10.3390/ijerph191711133 (PMC9518022; doi:10.3390/ijerph191711133)
Supplement: Supplementary file 1 [file ijerph-19-11133-s001.zip › ijerph-1893482-supplementary.pdf]

Supplementary material

Table S1. The correlation matrix (non-parametric) of candidate self-presentation items

Item	1	2	3	4	5	6	7	8	9
**1**	1.000								
**2**	0.665 **	1.000							
**3**	0.619 **	0.869 **	1.000						
**4**	0.444 **	0.598 **	0.599 **	1.000					
**5**	0.360 **	0.385 **	0.381 **	0.397 **	1.000				
**6**	0.199 **	0.170 **	0.182 **	0.237 **	0.219 **	1.000			
**7**	0.406 **	0.479 **	0.477 **	0.402 **	0.288 **	0.179 **	1.000		
**8**	0.510 **	0.600 **	0.584 **	0.478 **	0.339 **	0.204 **	0.631 **	1.000	
**9**	-0.83 **	-0.139 **	-0.140 **	-0.099 **	-0.022	-0.060 **	-0.019	-0.074 **	1.000

Note. Item 1: «I use a lot of time and energy on the content I post on social media»; item 2: «It is important to me that my posts receive many likes and/or comments»; item 3: «It is important to me to have many followers on social media»; item 4: «I delete posts on social media that do not receive enough likes and/or comments»; item 5: «I retouch pictures of myself to look better before I post them on social media»; item 6: «It's easier to be myself on social media»; item 7: «What others post on social media (images/status updates/stories) makes me feel less content with myself and my life»; item 8: «The response I get for what I post (images/status updates/stories) impacts how I feel»; item 9: «I don't care about how many likes or comments I receive on social media»

**Correlation is significant at the 0.01 level (2-tailed)
